# Women, Addictions, Mental Health, Dishonesty, and Crime Stigma: Solutions to Reduce the Social Harms of Stigma

**DOI:** 10.3390/ijerph21010063

**Published:** 2024-01-05

**Authors:** Sarah Page, Sophia Fedorowicz, Fiona McCormack, Stephen Whitehead

**Affiliations:** 1 Centre for Crime, Justice and Security, Staffordshire University, LW126 Ashley 2 Building, College Rd., Stoke-on-Trent ST4 2DE, UK; 2 Expert Citizens CIC, Federation House Station Road, Stoke on Trent ST4 2SA, UK; sophia.insight@expertcitizens.org.uk; 3 Centre for Health and Development, Staffordshire University, LW126 Ashley 2 Building, College Rd., Stoke-on-Trent ST4 2DE, UK; fiona.mccormack@staffs.ac.uk; 4 Independent Researcher, Centre for Justice Innovation, 102, Edinburgh House, 170 Kennington Ln, London SE11 5DP, UK; swhitehead@justiceinnovation.org

**Keywords:** women, addictions, drugs, alcohol, stigma, social harms, trauma, social work, police, non-judgmental, sexual misconduct

## Abstract

British drug policies could underserve women with treatment needs, and this paper provides evidence that communication through the words and actions of professionals across drug and alcohol services, health and mental health, social work and the criminal justice sector can leave women feeling stigmatised and failed. Women live with the stigma of ‘the lying addict’; however, documents and courtroom statements provided by professionals can misrepresent women’s experiences, which exacerbates social harm. Data are drawn from feminist participatory action research, where female lived experience experts worked alongside academics to implement a qualitative study using interviews and focus groups with women using treatment services (n = 28) and an online world café with professionals working with these women (n = 9) and further professionals providing support at lived experience data collection events (n = 5). This data set is cross-referenced with one-to-one and small-group interviews with professionals in the field (n = 17) conducted by a third-sector partner. Findings establish that stigma negatively impacts the identification of treatment needs and access to timely and appropriate service delivery. Social harms to women with addictions could be significantly reduced with timely, authentic, honest, gender-informed and trauma-informed practices for girls and women using drugs and alcohol to self-medicate from traumatic experiences.

## 1. Introduction

The ‘From Harm to Hope’ (2021) British drugs strategy [1] continues an abstinence trajectory, including aims of delivering quality recovery treatment and reducing stigma, following Black’s [2] recommendations. Black [2] requested commissioning changes, increased treatment funding, capacity building and greater accountability, and improved access and delivery of enablers of recovery, such as housing and employment services. The drug strategy [1] briefly mentions women’s differing treatment needs without nuance or associated service delivery guidance [3]. Addressing substance use issues in the UK is a high priority, with increases in drug-related deaths [4], increasing drug-related violence and societal costs exceeding £19 billion per year [2]. Also of concern are alcohol-related mortalities [5,6] and associated taxpayer costs of over £14.5 billion per year [7]. Many drug users also use alcohol [8], and both are a public health concern [9]. Government figures from April 2021 to 2022 indicate 289,215 adults accessing drug and alcohol services, and 33% were women [10]. Whilst the drugs strategy [1] focuses on illicit drugs, it mentions alcohol, and for this paper, we extend to both, using the term ‘substance’ interchangeably for alcohol and/or drugs.

Some drug strategy approaches [1] contravene international research findings, including tougher sentences for recreational users [11]. Punitive approaches can deter people from treatment; for example, state child removal fear is a barrier to women seeking help for alcohol use [12], and child removal can trigger increased substance usage [13,14]. Structural issues can exacerbate substance usage [15,16], and Link and Phelan [17] identify a power requirement to stigmatise others, for example, social, economic, and political power. Ultimately, punitive drug legislation creates ‘state-driven stigma’, disproportionately harming women, including violence and incarceration [18]. Furthermore, increasing punishment for recreational drug users would contravene the Female Offender Strategy [19] aims of reducing incarceration, with acknowledgement of the interplay between women’s offending, substance misuse, homelessness, mental health, and trauma experiences.

In Goffman’s (p. 4, [20]) view, addiction and mental health fall into the category of ‘blemishes of individual character’, resulting in stigma leading to discrimination by standard setters and those abiding by societal norms. Drug users experiencing stigma are acknowledged in underpinning drug strategy research [2]; however, the intersection between gender and stigma is not addressed. For example, women utilising drug and alcohol services experience stigma more acutely when pregnant, child rearing or if engaged in sex work [21]. ‘Sexual stigma’ attributed to women engaged in sex work and sex trafficking is socially constructed at the macro institutional and political levels [22]. Macro-level occurrences have implications for the micro level, and Addison (p. 298, [16]) articulates that stigma is ‘done between people’, resulting in social harm experiences. Stigma is evident in the actions of others, including male service users targeting female service with previous sex work experience to initiate exploitative relationships to fund their substance use [3,23]. Exploitative ‘survival sex’ and sex work also occur for homeless women with multiple needs, including drugs, alcohol, and mental health [24]. Homeless women experience multiple stigmas [25], which are likely exacerbated by the contravening of the woman as a homemaker gender norm [26].

Exploitation is further noted with women and girls from looked-after care and/or domestic abuse victimisation backgrounds being groomed into drug and sex trafficking [27]. Males are stakeholders in females remaining in active substance use [3], which could extend itself to what is described as ‘power stigma’ [28]. Coercion negatively impacts mental well-being [29], and female domestic abuse victims using substances experience Post-Traumatic Stress Disorder symptoms more acutely, including dysregulation of emotion [30]. International research has identified that brain injury from domestic abuse can impact short and long-term psychological and cognitive functioning [31], a similar picture for those with adverse childhood experiences [32,33]. It can be argued that if neurological damage from brain injury is repaired, vulnerability to future substance usage might be reduced [33].

Child rearing [21] and child removal increases stigma for women who use substances [13]. Child state removal negatively impacts substance-using mothers, often exacerbating substance usage [13,14]. State removal is six times more likely for substance-using mothers than fathers, indicating a gender inequality [34]. Mothers tend to be in poverty, young, have poor mental health and poor housing [34]. Improving mental health and suicide prevention support could help reduce child removals [34]. Broadhurst and Mason [14] advocate for mandatory post-child removal court proceeding support to mothers as a cost-effective intervention, given that rehabilitation would support future childbearing and child-raising. Furthermore, better state policies pertaining to addressing poverty would reduce the likelihood of child removals per se [35]. Whilst safeguarding children is incredibly important, it seems apparent that UK policies and practices are not fully effective and can lead to further harm, including stigma, sexual exploitation and crime engagement.

Corston identified prison professionals responding to women in stigma-inducing ways through empathy deficit [36]. More broadly, women experience criminal justice gender inequalities for breaking gender norms [37]. Rutter and Barr (p. 179, [38]) posit that females not living up to the ‘idealised whitesupremacist, heteropatriarchal, neoliberal constructions’ of the ‘good woman’ and ‘good mother’ experience shame and stigma. Essentially, compassion has been lacking towards women who use substances by the services endeavouring to rehabilitate them. This is of particular concern because female drug and alcohol dependency is mostly a response to trauma and poor mental health (p. 459, [21]), and these factors contribute to offending behaviour [39]. Rutter and Barr [38] highlighted that criminal justice staff working with female offenders to reduce feelings of shame and stigma are ultimately facilitating crime desistance. Females seem to be coerced into crime [40,41], and crime can also be a form of agency [40]. Male drug users who perpetrate domestic abuse have acknowledged coercing partners into substance usage and crime [41].

Black’s [2] report requested further research into effective drug treatment provision. Whilst the sociology of health literature highlights addiction as either a disease model or a social model (p. 23, [42]), both medicine and talking therapy can assist people in recovery [3,43]. However, with national numbers of substance users increasing, including women who use drugs [21], what is currently on offer in the UK is clearly not a panacea. Acknowledging neurobiological dimensions of addiction regarding the brain disease model is thought to reduce stigma because professionals need to employ evidence-based treatment and dismiss the notion that substance usage is due to an individual’s moral failing [43]. However, others argue that this stance and claim of stigma reduction is questionable [44]. When purely a medical response is given, other issues can emerge, for example, drug dependency from women being given medical prescriptions for mental health issues stemming from ongoing domestic abuse victimisation [45] and in such cases, medicine does not address the social root issue. Whether the reader subscribes to the social or disease model, what is agreed upon is that drugs and alcohol impact both the body and the brain [3,46]; as such, holistic treatment is welcomed.

The main purpose of our research has been to undertake primary qualitative research to gain a more nuanced understanding of women’s lived experience of community treatment provision. This paper is focused on stigma-induced injustices that occur in relation to interactions with others, including a range of professionals. We also consider how stigma is multi-layered and multi-faceted. Ultimately, stigma impinges on women accessing trauma-informed and gender-responsive service provision, and we provide some solutions for reducing stigma.

## 2. Materials and Methods

A participatory methodology was utilised, enabling a more democratic approach drawing on strengths from a range of people [47,48]. Female academics from Staffordshire University collaborated with an undergraduate student, staff and members of a third-sector lived experience-led organisation (Expert Citizens CIC) and a third-sector innovation organization (Centre for Justice Innovation) to co-create and implement qualitative research. We aimed to garner insights into women’s experiences of accessing community drug and alcohol treatment and associated professional experiences. For data collection with women with lived experience of drug and alcohol services, a feminist approach intertwined with participatory methodology, with an all-female research team conducting interviews and focus groups with women in recovery (n = 28) who were accompanied and supported by female professionals (n = 5). A feminist approach was selected due to our understanding that women may have experienced drug and sex trafficking grooming predominantly by males [3,23,24,27]. Females researching with females reduces the power dynamics for the researched [49,50], with recognition of the global experience of ‘oppression and exploitation’ that women encounter (p. 4, [51]). Lived experience experts co-producing research questions and co-facilitating data collection alongside academics further reduce power dynamics [51]. Characteristics of feminist research include innovations to research methodology, reflexivity and prioritising political change ‘over procedural, epistemological and disciplinary orthodoxy’ (p.13 [52]). Both feminist and participatory researchers aim to understand oppression and work to address the issues [51].

With there being a high possibility of traumatic experience disclosure, we offered women the choice between an individual interview or a focus group. Focus groups are viable for discussing sensitive health issues [53], and women engaged in a therapeutic peer-support group may want to attend a focus group with peers rather than navigate an individual interview. A female and a male researcher from a third-sector partner organisation (Centre for Justice Innovation) conducted one-to-one and small-group interviews with professionals in the field (n = 17). The final data collection phase with professionals (n = 9) was via an online adapted world café event led by academics and supported by the wider research team. World café is a participatory methodology that allows for cross-referencing data and the co-designing of solutions [54].

The British Sociological Association’s [55] ethical guidance of voluntary involvement, informed consent, anonymity, appropriate questioning, not harming participants, debriefing and data protection were employed with ethical approval granted by Staffordshire University. Furthermore, researchers who had prior research training were recruited to the project, and everyone was renumerated for their contribution.

A mixture of purposeful, convenience and snowball sampling was used to reach women with lived experience and professionals. Primary data collection was undertaken in three stages (see Table 1). Relevant stakeholders received email invitations from our funders via existing networks to attend an online meeting about the research. This helped professionals determine whether to voluntarily engage in the study either directly or to be a champion for linking women in their services to our study. Four community sector organisations agreed to facilitate data collection on their premises with women with lived experience.

Remuneration consideration is important in participatory research [56]. We renumerated organisations for hosting data collection events on-site and providing additional support. Women with lived experience were given a £20 gift voucher in appreciation of their contribution to the study. In-person data collection occurred in therapeutic rooms; however, one woman requested a telephone interview with an academic researcher and received an email gift voucher. Interviews and focus groups were audio recorded and transcribed verbatim. Co-produced question sheets allowed for semi-structured interviewing. For interviews with women with lived experience, questions included the following sample:

- Have you ever been told that your needs were too complex for the service? Or had unmet needs?
- What barriers were there to you getting the support you needed?
- Did the service that you accessed cause you any trauma/harm/upset?
- When you have had contact with blue light services (police, ambulance, fire)—what was good about the interaction and what could be improved?

Similar questions were used in the lived experience focus groups, which started with an icebreaker of using four cartoon pictures representing the social harms of intoxication (sickness, embarrassment, injury/violence and sexual interactions) and asking women to share what resonated with them. Interviews with professionals included the following questions:

- Based on your experience, what are women’s experiences in seeking to access substance misuse treatment?
- Based on your experience, what are women’s experiences of undergoing substance misuse treatment?
- What are the key characteristics of effective substance misuse treatment for women?

Professionals engaged in the online adapted world café data collection event were asked whether lived experience findings resonated with their work and what further information they would want to share, including solution ideas for addressing women’s drug and alcohol treatment needs more effectively.

Data collected by academics and lived experience experts was inductively processed, using reflective thematic analysis [57,58] and conducted by an academic and lived experience expert, with wider team sense checking via meetings, email exchange and informally during data collection commutes. Sense checking in participatory research is helpful with academics leading the analysis and dissemination work [24,48]. Data collected from the Centre for Justice Innovation was deductively processed using themes identified from initial secondary research. Reflexive thematic analysis was selected (over, for example, the positivist approach of coding reliability) because of its fitness with the qualitative, participatory and feminist research underpinnings. As discussed by Braun and Clarke [58], data are both situated and contextual, with researcher subjectivity used as a vehicle for knowledge production rather than a ‘threat to credibility’. Fundamentally, themes are the final ‘outcome’ of the researcher’s coding and theme development, and as such, the researcher plays an active role in the analytical process [58]. Online meetings between the academic principal investigator and Centre for Justice Innovation third sector manager led to the finalisation of project-wide themes for stakeholder reporting [59]. For this paper, keyword and prior coding searches ascertained data, and the principal academic investigator amalgamated data sets identified new stigma-related insights.

## 3. Results and Discussion

Wider themes related to adverse childhood needs and domestic abuse- influences on drug and alcohol usage, child state removal and bereavement impacts, the disparity in trauma-informed understanding and application to practice, the need for joined-up assessments, appointments and practices between key service providers to ensure holistic needs are met and the potential for co-location of services, the challenges of accessing mental health treatment and criminal justice treatment and continuity of care post punishment [59]. This paper focuses on the stigma pertaining to women dependent on substances. Women reported feelings of shame induced by interactions with close family and friends, and associated stigma was reinforced through negative interaction with professionals. Women identified appearance decline and cringeworthy intoxicated behaviour as creating further shame. Women and professionals noted that poor professional practice linked to stigma perception reduces access opportunities to services and effective and timely interventions.

### 3.1. Stigma, Relationship Tensions and Exploitation, Appearances and Cringeworthy Behaviour

Women viewed pictures showcasing four social and health in-toxification harms (a face with kiss marks entitled ‘oooohh’, a face portraying sickness entitled ‘bleurrgh’, a bruised face entitled ‘aarrgh’ and a face depicting embarrassment entitled ‘cringe’). Women identified with them all, and the ‘cringe’ picture led to stigma insights:

‘*Mine’s cringe as well, erm, I cringe when people and family tell me what I’ve done (SP—ok) I really, really cringe and think “how on earth could I have done that or said that?” Erm, yeah, it’s cringe. I cringe at meself when I think about things…*’

(FG2P7)

Comments reflect what Addison (p. 308, [16]) refers to as stigma-induced ‘ugly feelings’. Women reflected when alone and with others, including watching CCTV footage with police officers. Repetitive ‘cringe’ conduct seemingly led to internalising stigma and increased shame, including shame regarding the diminishment of physical appearances:

‘*… lots of drunken injuries, erm, and my hair was always a little bit of a mess (laughter). I stopped taking care of myself really, so that’s me…*’

(FG2P12)

Physical appearance decline validated the sense of failure, which suggests the ‘good woman’ stereotype described by Rutter and Barr [38] extends to include being well-presented. Women linked physical appearance with addiction identification:

‘*… there is still a stigma… a lot of us in this room… when we were drinking thought “no, I’m not an alcoholic”, because I’m not on a park bench, I’m not filthy dirty, you know, I don’t eat out of bins, that’s an alcoholic…*’

(FG2P8)

Women acknowledged that appearance judging was ‘shallow’ because ‘rich’, well-presented people also use substances. Goffman [20] highlights that appearance can be a feature in being stigmatised, and our findings concur with this despite the recognition that such judgements are deceptive.

Unequal relationship dynamics based on appearance, age and gender were witnessed regarding male service users exploiting females through sex work and theft to fund substance use. As such, we posit that women were experiencing what Link and Phelan [28] refer to as ‘power stigma’, with men proactively keeping women dependent on substances for male sex and drug use gratification. Power stigma seemingly interplayed with ‘sexual stigma’ and ‘sex-trafficking stigma’ [22]. Power stigma was also apparent in longer-term interpersonal relationships that had dissolved, but family court disputes continued:

‘*… they’ve used alcohol to cope with the abuse. Then when it has come to family courts, they look like the ones that have got an addiction, so the kids remain with the father and the women are looking like they can’t get a grip on their dependency.*’

(MH Specialist 1)

Women were blamed for inappropriate behaviour while their abusers continued to punish them, with children put at risk due to professionals lacking understanding. Noteworthy is that women used substances as a coping mechanism for their adverse childhood experiences and were often introduced to substances by a family member, boyfriend or friend. As such, substance use was often because of coercion or in response to coercion.

Women tended to experience isolation or ongoing difficult relationship dynamics with people who regarded their behaviour as problematic. This seemingly pushed women into relationships with other drug and alcohol users, making recovery more challenging, sometimes influencing decisions to move locations to avoid drug dealers and users. However, this also left women isolated and vulnerable:

‘*… you can be isolated when you move away from your circle of drinking or drugs because it’s all you ever knew and then when you isolate, it’s not a good start to your recovery, to isolate.*’

(FG2P4)

Women had time to reflect on life course events when isolated, which may increase feelings of shame. Becker [60] described cannabis users relating more to a using subgroup than to non-using family and friends, partly to avoid ‘labelling’ consequences. In our research, relational detachment was initiated by the woman, by social services or via a loved one’s choice. Multiple disadvantages incorporated multiple stigmatisation and relational breakdowns, particularly the combination of substance use and mental health. This was experienced in unpleasantness akin to ‘persecution’ detailed in Karpman’s triangle [61].

‘*… basically that I’m no good… especially having mental health as well, it’s a double whammy, erm, it’s like a stigma really, er, around, um, mental health and alcohol addiction because it does actually come together… to be honest with you, err, people weren’t nice to me at all, even my friends.*’

(InterviewP22 Woman)

‘*My daughter’s very judgy of me and what I do, but she does balloons. But all the kids do it so it’s like…*’

(FG2P2)

Persecutors included families using substances recreationally, whereas dependent use was less acceptable. This was depicted as ‘…a hierarchy’ to drug use. However, some professionals surprisingly did not appear to understand this hierarchy:

‘*… they have got quite traumatic pasts… started using substance to cope with those feelings... I’ve had quite a few marijuana users recently… the views on weed, cannabis, marijuana… a lot of my women see it as it’s natural… almost acceptable… They don’t see drug misuse in cannabis in the same way they would see drug misuse using heroin, which is quite odd really.*’

(MH Specialist 5)

Cannabis is somewhat normalised in British society despite being illegal [62], and our findings indicate that Becker’s [60] ‘outsider’ research is more attuned to heroin, cocaine, or alcohol dependency rather than cannabis usage. The ‘smack-head’ heroin user feeling the ‘lowest of the low’ features in Addison’s [16] findings and is echoed in Radcliffe and Stevens [63] study in England, which found other drug users felt uncomfortable using drug services known for ‘junkie’ engagement. Best (p. 198, [64]) indicates drug addiction as slightly more stigmatised than alcohol in world rankings, a finding notable in our study. We also identified a hierarchy pertaining to whether a woman had been incarcerated or not, with more stigma associated with those engaged in the criminal justice system. In both the substance use and offending hierarchies, we found prejudice dissipated as women identified with one another’s shared trauma experiences.

‘*…That’s the thing with a hub like this… I get where you are coming from… I never dreamt of speaking to somebody like that [referring to a heroin user] about another addiction… it is interesting to see all different angles and all walks of life…*’

(FGP3)

‘*… they were scared to say that they’d be in jail… I was like “we are all in the same”, “we’re all here cos we’ve been in trouble one way or the other, whether you’ve been to jail or not” (agreement from the group). We don’t look down our nose at you…. There might be a stigma there…*’

(FG1P1)

De Silva’s Brazilian study [65] found that homeless sub-groups required drug usage for membership and enabled protection from societal stigma; essentially, peers were a forcefield to stigma. Whereas our findings suggest women are not protected from stigma through forming support groups, they are able to process stigma by talking about their experiences with peers and supportive professionals. Essentially, group recovery connections enable ‘pathways to change’ (p. 45, [64]), and positive connections are necessary with family, peers, the community, and professionals to achieve this (p. 198, [64]).

### 3.2. Stigma from Professionals, Judgement and Gender Stereotyping

Professionals reflected on circumstances that led to women being referred or self-referring to treatment. They noted that females seemingly accessed services as ‘a last resort’, and this may be due to gender stereotyping [26] regarding family roles:

‘*I wonder if it’s because that the role of a female in families and caregivers… means that people try to cope. Because of stigma, …and everything else, is that why we see people at the last possible moment before things go really pear-shape?... so they’re presenting in need somewhere else in crisis, and then they’re being directed here or pushed here, as well.*’

(Professional P4)

As such, the ‘good mother’ and ‘good wife’ stereotypes [38] created a barrier to women feeling able to talk about their issues. In women-only recovery groups, women felt more able to disclose issues and obtain support. Women reported empathy from peers and professionals, and professionals with lived experience had advanced empathic responses and were inspirational role models.

‘*The way that the staff treat ya… I’ve been here a year, I come most days, I’ve not had anybody judge me. (group agrees). When people judge ya, it’s like they make you put yourself down don’t they?...*’

(FG3P1)

Feeling judged led to stigma internalisation. A key factor in not feeling judged was workers being empathic, authentic and displaying warmth, akin to the core conditions described by Rogers [66].

Researcher: *And when they do the ‘friendly’ rather than the ‘I’m an official’, they are the things that help?*

FG1P1: *Yeah, makes a hell of a difference because it makes you actually feel like you know what, they’re not just doing this as a job, they’re doing it because they want to.*

FG1P2: *Like they really care, it’s not an act because it’s their job.*

Conversely, women inferred some professionals worked purely for pay rather than a genuine desire to help people. This could be experienced as a ‘Power Stigma’ with professionals needing women to remain alcohol and drug-dependent to keep employment. Poor practice could also be due to austerity measures impinging upon sector capacity building [2].

### 3.3. Discriminatory Practice, Language, Mistakes and Misconduct

Professional language and official terminology added to the stigma. One woman described her husband initiating a ‘non-molestation order’ due to her being intoxicated and putting a window through on the house she was a joint owner of but did not live in. She found the term ‘non-molestation’ painful; it sounded like she had abused someone rather than damaging her own property. The term ‘addiction’ was also perceived as problematic because it creates ‘othering’, which concurs with reflections from Page, Bratt and Oldfield [3]. Professionals were also distressed by discriminatory language used by other professionals:

‘*We still hear statements from the police. You know, if we’re trying to support a woman who’s involved in sex work to report a rape, we still hear sentences like, “Oh, well she was asking for it.” Or… “It’s part of the job.”*’

(CJS Specialist 2)

Here, we see ‘sexual stigma’ [22] being expressed by the police professional. Instead of professionals being empathic, there were accounts where professionals blamed women. For example, in a case where a local authority manager refused to re-house a woman due to the woman returning to her abusive partner:

‘*…we set up… a professional’s team, so we’d have the police, probation, the council, the drug service, and the street outreach team that I was on. We were looking after this one girl… she was using crack, she was pregnant … and she was in a DV relationship. And we rang to get her an emergency move… the person at the council, her words were “well it’s her own fault because she keeps going back to him”…*’

(FG3, worker)

Essentially, the manager was not offering ‘unconditional positive regard’ as described by Rogers [66] and had unrealistic expectations rather than compassionate understanding regarding the multiple layers of challenge that this woman was experiencing. It seems apparent that this manager lacked knowledge of the decade of research that explains why women return to abusive partners [67]. Evidence-based practice advocates for women to be safeguarded via meeting accommodation needs due to increased vulnerability to abuse and exploitation when homeless [24]. Many women in our study disclosed domestic abuse victimisation, including head injury, and it is surprising that there was no mention of referrals to head trauma injury specialists. Mental health referrals occurred with significant delays in assessment and treatment; however, physical impacts on the brain were seemingly not attended to.

Women were also privy to judgmental comments made by professionals, which ultimately led to women receiving poor-quality treatment or women not being able to access relevant services and provisions. Addison (p. 309, [16]) refers to the ‘mechanisms of stigma operating in interactions with service providers’. We found that a broad range of professionals expressed stigma-inducing responses to women. On occasion, women were essentially communicating un-met trauma needs through aggressive communication and were not responded to in trauma-informed ways:

‘*…they do get aggressive sometimes when they turn up at the hospital and they can get frustrated… the only way that they can get their point across would be to shout… so they’re asked to leave… sometimes security are called, sometimes police are called… So, then they’re out in the cold… in withdrawal…*’

(CJS Specialist 6)

Service exclusion resulted from professionals not seemingly understanding communication as a form of trauma expression, and instead, professionals seemingly regarded the women as ‘undeserving patients’ (p. 309, [16]). Instead of ‘rolling with resistance’ and empathic motivational interviewing responses [68], some professionals were intolerant. Women and professionals indicated some Accident and Emergency staff refused to provide a service when women were intoxicated or delaying access to treatment. Experiencing ‘silence’, ‘long waits’ and ‘inadequate note taking’ instead of support is essentially a passive-aggressive response [69]. Women also talked about long waits when in the hospital and overhearing professionals making judgmental comments:

‘*As soon as you say you’re drunk,… they [ambulance professionals] said they won’t take you if it’s alcoholism… So yeah that’s another thing, you’ve gotta sober up before you call the ambulance cos they won’t take you.*’

(FG3P3)

‘*…when you’ve been in and out of the hospitals, they know you… (P3—They get sick and tired of you don’t they?)… they stand at the bottom with the curtains closed and they’re talking about you, “oh she’s an alcoholic, she’s been in here”… You hear it so many times and then they treat you like that (P3—yeah)*’

(FG1P4 and P3)

Repetitive service contact whilst intoxicated or when in a state of drug or alcohol withdrawal seemingly elicited increased levels of stigma from professionals. Women experienced being emotionally and verbally beaten up by professionals and were also physically beaten up by police officers:

‘*…I’ve been beat up by police officers plenty of times… they think they can do whatever they want… they’re meant to have… some kind of trauma training… I got arrested so many times before I went to prison and all I needed was someone to care a bit more and actually help me… I had mental health, bad mental health that had been undiagnosed since I was a child… And they’re there just tarnishing me, thinking I’m just a criminal, when really, I was just a mum that needed help… They’re just not understanding, I hate the police.*’

(FG3P2)

Women elaborated that mostly male officers violently arrested and detained them, and some women initiated or reciprocated the violence when intoxicated and in a state of fight or flight adrenalin arousal. Women were surprised that police officers were not implementing trauma training assumed to have been undertaken. One professional also wondered whether police capacity was stretched, impinging upon trauma-informed approaches being implemented. However, one police force was known to be referring women to treatment as an out-of-court order rather than initiating criminal justice punishment proceedings. This force asked treatment providers about times to avoid interviewing female victims of crime so that substitute drug collection remained viable. Whereas other police forces and officers evidently failed to establish and attend to what was going on in the lives of women they regularly arrested, and a repetitive cycle of offending and substance usage ensued.

Earlier trauma-informed interventions and appropriate treatment referrals would likely reduce the revolving door phenomenon. Professionals wanted earlier interventions to reduce women reaching crisis points and entering the criminal justice system. The revolving door of people engaging, leaving, and re-engaging in treatment services was problematic to organisational capacity. Furthermore, women did not always want to see previous support workers due to feeling labelled by them.

‘*… I feel uncomfortable because while she was off [drug support worker] I had my other drug worker then, who knew me when I was on drugs… So, I felt like her barrier were up straight away cos obviously she’s not gonna believe that I’ve changed sort of thing… It’s one of them innit (pause). I’ve just gotta be strong for my daughter, you know. It’s all you can do, innit?*’

(Interview P2)

Women felt written off by professionals, and this was further confirmed when professionals did not provide support or further recovery opportunities. This was particularly apparent with social workers initiating child removal:

‘*… my social worker was possessed by the devil… (P2—ain’t they all?). She was acting so unprofessional, like any means to take them kids (P2—yeah)... She made it very clear that she couldn’t care less about me… I don’t think it ever occurred to her how her actions affected me… she just lied a lot (Researcher—ok)… they did an unexpected visit and found me smoking a cigarette outside… she wrote down that she saw me coming in, fresh face of makeup, fully dressed with shopping bags… to give the impression that I’d left the children… Obviously, I hadn’t gone anywhere, I had morning breath and pyjamas… when they’re trying to get the kids off ya, they will tell whatever lies in front of the judge, in legal documents and it’s basically your word against theirs and they’re the professional so.*’

(FG3P3)

Women explained that child removal was incredibly traumatic and often resulted in increased substance usage to cope with the grief, which concurs with existing findings [13,14]. Women felt they had failed their children, which induced shame. The women did not specifically talk about whether their actions warranted child state removal; however, some talked about safeguarding needs regarding their violent partners. They also talked about domestic abuse victimisation perpetuating their drug or alcohol consumption. Best practice in social work advocates for women to have continued social work support in accessing alcohol and drug services post-child-removal court confirmation as a preventative measure for future children needing removal [14]. However, social workers were not perceived as a trustworthy source of support and based on the women’s accounts; best practice did not seem to be employed. After women had lapsed in response to the loss of the child(ren), much later, they went on to regard the potential to see their children again as a recovery driver:

‘*I’d like to see my kids again. I got this profound fear of dying and never setting eyes on them again.*’

(FG3P3)

‘*…that is literally what drives me every day, is that when they [my children] do come back. I have to be the best version of me that I ever was and they have to see that them going was the making of me…. Turn that pain into something kind of purpose.*’

(FG3P2)

Many of the women showed resolve to turn around situations. Where professionals encouraged women to make positive changes and provided empathetic and visionary support, women seemingly made progress. Meanwhile, professional dishonesty and inaccuracy are featured in women feeling stigmatised. In some instances, women challenged inaccuracies, and one woman experienced vindication but no apology.

‘*They [referring to drug support workers] try and help, but they’re not in my good books… I was in a conference, a core group for a conference. The first time I’ve had my daughter and… they was like “I’ve used, I haven’t got my script, I haven’t done this…” and I said “no, that’s not correct” cos I was pregnant for a start… Well, they haven’t even said sorry. They just said it was our mistake, but that’s not good enough…*’

(IWLEP2)

This woman also noted the double standards of how professional mistakes were somewhat overlooked, whilst her mistakes had instant ramifications. In this scenario, her child could have been taken into care, and she worried her social worker would be increasingly suspicious of her despite her being vindicated. She stated she had not used heroin for ‘*three years*’ and was still having to undertake urine tests to prove her ‘drug free’ status and child-rearing suitability. Professionals requesting urine samples can be indicative of power stigma. Professionals making such mistakes were not necessarily common practice, but when it did happen, the women experienced emotional distress and, in some cases, harm associated with the repercussions.

In other situations, women felt professionals were not making mistakes but were being intentionally dishonest, and this could be interpreted as a form of punishment for the women. For example, a woman mentioned purchasing a Taser gun and moving location because her ex-partner threatened murder. She was arrested upon the police finding her in possession of drugs and a Taser gun. The police statement did not concur with her version of events:

‘*…What they wrote in the statement was all lies… They said they found drugs in the car and that but they didn’t. I had amphetamines on me, which I gave in at the desk, so they didn’t find it at roadside. They said I tried to blag them and tell them the taser was a torch and it wasn’t, but I was honest from the beginning…I’m not a big fan*’

(FG1P1)

Male professionals, albeit a minority of male professionals, also misused their trusted positions to gain sexual gratification and were somewhat protected by a system that validates professional accounts above accounts by women who use substances. In the UK, male police officer sexual misconduct has been typically directed towards female victims and witnesses of crime, with less commonality when victims had poor mental health and/or used substances, although this is likely to be an underreported issue [70]. However, female officers were more likely to engage in sexual misconduct with offenders [70]. According to Open Access Government [71], there is a Met Police investigation into sexual misconduct at present. There is also a national healthcare call to investigate male healthcare professionals regarding sexual harassment towards female colleagues with concerns about patient sexual safety [72]. One woman in our study said:

‘*… I got arrested, erm, and I seen the police doctor…let me take however many drugs I wanted to… then said he needs to examine me and sexually assaulted me. I then [physically] assaulted him and I told the police about it, and they just let me out, they just got rid of me… it’s all on files now… I happened to google his name and he’s in prison for life now. He did it to four other prisoners and he raped children… I used to think “have I made that up? Did I dream it? Did I get wrong?”… then I started working [name of hospital omitted] and then there was a doctor there who knew about my addiction… he would like sort of say “right, ok you’ve got to suck my willy” or ‘you’ve gotta sleep with me”… “If you tell people, you’ll lose your job”*’

(InterviewP3)

Romo Perez [73] highlights that police culture can include covering up for colleagues engaged in sexual misconduct with offenders, and there is an assumption from officers that the woman is the sexual initiator. In our research findings, it was two medical professionals initiating sexual misconduct, and one was a male police GP, whereby the police officers overlooked the offence and did not follow up on the original complaint. Another woman emphatically said she would not let a male medic examine her as part of her treatment requirements, and this may have been for similar reasons. Professionals articulated challenges in recruiting female medical staff to work in community alcohol and drug treatment services, and this leads women to be more vulnerable to sexual abuse. Training and recording processes for complaints are needed in the health sector [72], and we found this is likely to be needed in police forces.

Sexual conduct abuses, physical assaults and stigmatising behaviour from professionals communicated disrespect and dislike to the women, and these feelings became reciprocated due to stigma-influenced injustice. Unsurprisingly, this created a barrier to women seeking help or reporting crime.

‘*… so many women in this room say “we don’t trust the police”, “we can’t ring the police”, and “we can’t ask them for help on anything”. Imagine how many women are out there… going through what they’re going through right now in their homes and they can’t call the police cos they don’t trust the police. It’s wrong.*’

(FG3P4)

Furthermore, women perceived the police as stigmatising women based on gender, addictions, and heritage. Multiple stigmatisations created multiple layers of harm and barriers to accessing services.

‘*… it makes a difference what colour you are. (P2—it definitely does)… I would never call the police on any black man [even an abusive partner]… in my community (a) there is a justified mistrust of the police and (b) you’re treated differently as a black person…*’

(FG3P3)

Women felt justified in their distrust of the police based on police behaviour that they had personally experienced, witnessed or heard about within their immediate community.

### 3.4. Professionals Having Low Expectations and Setting Women up to Fail

A further barrier to accessing services is also presented in professionals requesting multiple appointments at different places:

‘*… after staying in to do the whole of my licence so I can go home and be free with no probation… because they don’t do anything for me… So, I’ve gotta go all the way over there, cos they’re thinking… “she isn’t gonna come over here, so we might as well just get the papers ready for a recall right now”. It’s like they’re setting me up to fail, ain’t they?*’

(FG3P4)

Women felt set up to fail by professional expectations. Women were trying hard to keep to professional agendas to ‘stay clean’ and desist crime. They kept themselves in check on telling the truth to professionals and regularly made comments like ‘honestly’ when they referred to situations to reinforce that they were truth-telling. Evidently, the ‘lying’ stereotype that Kemp [74] associates with addiction had impacted the women who were called out by professionals and one another for ‘*lying*’, and yet some professionals lacked honesty, often leading to the women looking dishonest when they shared a different account of events. Whilst women in recovery did acknowledge previous lying through memory loss or to manipulate situations, they were endeavouring to be truth-tellers in recovery.

‘*…I have to tell the truth cos this medicine that I want to go on is serious medicine and I would not lie about it, you know… it works being truthful. When you’re in addiction, you lie… lied my socks off… not anymore, I’ve done enough of it…*’

(FG2P1)

Women explained that lies are often caught out and that, ultimately, you end up lying to yourself. So, they regarded honesty as an important component of recovery. Honesty with themselves and with others, including professionals. Not receiving the same level of what Rogers [66] regards as ‘transparency’ and ‘congruence’ from professionals was troubling to the women. When professionals were honest and reliable and went above and beyond for the women, it gained the women’s trust and was an enabler for recovery. Noteworthy was that there were more recovery support workers exemplified as providing good practice than those who were not, particularly support workers who went the extra mile for women, for example, in providing essential resources for the women or organising and encouraging the women when on annual leave, whereas healthcare professionals, social workers, the police and prison staff were acknowledged less for good practice and more for judgmental responses that increased trauma.

## 4. Solutions

Within the context of participatory research having a social justice outcome [48], we asked participants to share solution ideas and amalgamated these with reflections from the findings and the participatory process used in this research that valued the input of lived experience expertise. Broader recommendations pertaining to the value of women-only service delivery can be found in Whitehead, Page, Jeffrey and McCormack’s [59] report. The following recommendations relate specifically to reducing stigma:

- When women are working hard to be authentic, they want authenticity and transparency from professionals. Having greater transparency with what is recorded by professionals and an opportunity for service users to correct accounts, or at least have it on record that they disagree with the account, could build trust;
- Professionals demonstrating the core conditions of empathy, non-judgmental practice and congruence provide the right environment for women to feel safe to disclose issues and process stigma-induced-harms. Basic person-centred counselling philosophy [66] and skills would be useful for all professions associated with working with women who use substances (e.g., treatment, healthcare, social work, housing, prison and the police). Professionals adopting motivational interviewing practices of ‘rolling with resistance’ [68] would also support a more trauma-informed approach when women vocalise frustration at service providers;
- A mechanism to reduce hierarchical stigma pertaining to substance use and engagement in the criminal justice system was through women coming together and sharing their similar lived experiences; they were not alone on their recovery journey. Women-only therapeutic groups can help support women in talking about life challenges;
- Women welcomed lived experience experts working in recovery support worker roles because they enhanced the empathy that women experienced and provided role modelling and inspiration;
- Social harms to women who use alcohol and/or drugs, including stigma-related harms and engagement in the criminal justice system, could be significantly reduced with timely gender-informed and trauma-informed practices for girls and women who are using drugs and alcohol to self-medicate from traumatic experiences. The police adopting a trauma-informed approach to arrest and detention practices would be welcomed;
- Mandatory training with regular updates for professionals in all related services pertaining to trauma, gender discrimination and harassment, ethical professional practice, being non-judgmental, and responding to service user complaints is needed. Women assumed that professionals were trained to identify and respond appropriately to trauma, and this needs to become a reality. Including lived experience accounts in training is of paramount importance to increase professional understanding and empathy;
- Increasing the workforce, alongside allowing for reductions in workloads and increased supervision of practice, would be helpful for ensuring a trauma-informed approach is viable and misconduct is reduced. Combined with creating communities of practice with lived experience involvement.

## 5. Study Limitations

This study had a broad remit, and further nuances regarding stigma would be likely if the overarching project had specifically focused on this one element of women’s lived experiences of community drug and alcohol treatment services. A further limitation is in the transferability of findings to international contexts due to participants being based in the West Midlands in the UK. Wider UK research infers similar issues and experiences of stigma, which suggest that national transferability is applicable. Our sample size was substantial for a qualitative study, and we reached saturation on the core aim of the study [75]. It was beyond the scope of this paper to discuss the stigma associated with building signage and facilities that women interact with. Furthermore, more research is needed into addressing sexual misconduct practices and understanding the experiences of wider professionals to better understand the actions that they take, for example, child removal decision making, accommodation decline decision making and the neglect to refer women for trauma and brain injury treatment.

## 6. Conclusions

This study suggests communication through words and actions of close family and friends, alongside professionals, leaves women feeling stigmatised and set up to fail. Delayed action, inaction, non-communication, and inaccurate communication corresponded with women experiencing passive aggression from some professionals who seemingly perceived women as ‘undeserving’ of dignified and empathic service delivery. Power stigma was identifiable in that some professionals were perceived to be more pay-cheque motivated than authentically encouraging women in recovery through trauma-responsive practice. New insights include identifiable hypercritical practice of professionals accusing women who use drugs and alcohol of ‘lying’ or using substances, and yet some professionals misrepresent events in professional case notes, including legal documents for court hearings, which adds to the emotional trauma experienced by women. Such misrepresentations are further passive aggression and power stigma towards women, and they can have serious repercussions for women, including the potential for child removal. Greater transparency in recording information and providing women the opportunity to correct or to have it on record that they disagree with information could support trust building. A new contribution to knowledge is that women in recovery work hard to overcome the stereotype of the lying substance user and need to be taken seriously when they come forward with complaints regarding staff dishonesty and sexual misconduct. Sexual misconduct within police and healthcare settings is seemingly overlooked, and this needs further investigation. Our research calls for better trauma identification and response training, safeguarding and complaint recording measures, and supervision of practice across the sector.

Child removal is stigmatising and trauma-inducing, leaving women grief-stricken and feeling failure. However, women were able to find resolve for change in their desire to see their children again. Women also found their children were motivators for change when they remained the custodians of their children; however, they constantly had to prove themselves to professionals as ‘good enough’ parents. This included undertaking urine sampling despite being drug-free for some time, which is concerning professional practice. Such professional requests are indicative of power stigma. More research into professional practices is needed to glean greater clarity on urine testing, safeguarding procedures and appropriate referral pathways. Trauma and brain injury referrals for birth mothers and looking after children are needed. Presently, our findings are indicative that best practices are not being employed. Sometimes, this means that children remain in the custody of a domestically abusive father, which presents safeguarding concerns with recognition that the father’s behaviour has been a trigger for the mother’s substance usage.

Women experienced shame regarding their ‘cringeworthy’ conduct, including aggression toward professionals as a response to frustration and unmet trauma needs. Persecutory responses from family and friends added to women’s shame, and often, social distancing occurred. Stigma was reinforced and widened through negative judgmental interactions with professionals, including drug and alcohol treatment providers, social workers, mental health and health care professionals, the police and criminal justice professionals. New knowledge includes that professionals stigmatising women led to women feeling unsafe to seek help and were denied police investigation or medical response and intervention. Distrust and dislike became reciprocated as the women in our study resented professionals who disrespected them. Essentially, we saw a pattern of stigma widening and deepening, like ever-increasing circles with increasing intensity and internalisation. As such, stigma had a cumulative effect on women, particularly where multiple stigmas were apparent pertaining to gender, substance use, physical appearance, ethnicity, mental health, dishonesty, and criminal justice involvement. Findings suggest reforms are needed to policy and practice in Britain, which seemingly plays lip service to women’s treatment needs. When professionals adopted the core conditions of empathy, non-judgmental practice and congruence with women, as depicted by Rogers [66], women seemingly made progress. Professionals who went the extra mile for women were highly praised, whereas when professionals were not authentic, misrepresented women and/or were judgmental, women’s progress was hindered due to stigma-induced harm and injustices.

Despite stigma and challenging life circumstances, women demonstrated resilience and found solace in women’s support groups, which included peers, peer support workers and professionals. Such support enabled women to make positive changes, and the pains of stigma somewhat diminished as women discovered their experiences were shared by others. New insights include that stigma associated with the hierarchy of substances also reduced as women entered a shared recovery journey with peers and realised that first appearances do not tell the whole story. Lived experience engagement in service delivery provides a positive opportunity for improvements to practice and inspiration to women in recovery. Sadly, women felt that some professionals were not able to look beyond first impressions and stereotypes to understand what was going on for them as individuals, and as such, these professionals were unable to provide the necessary trauma-informed and gender-specific treatment approach that women realised was helpful for recovery.

## Figures and Tables

**Table 1:** Stages of Primary Data Collection.

Stage	Description	Participant Numbers and Characteristics	Researchers and Positionality
1	Individual and group interviews with professionals from third-sector services.	n = 17 (16 = Female and 1 = Male)	Conducted by a third-sector organisation (Centre for Justice Innovation). A female mixed heritage staff member with a postgraduate research qualification and their white male manager undertook interviews and analysis. This was peer research, with most working in the third sector.
2	This stage involved interviews and focus groups with women with lived experience of using drug and alcohol services. (a) Practitioners providing support at group events (2 were lived experience experts also counted in part b); (b) Interviews and focus groups with women accessing community services.	n = 5 (−2) (5 = Females) n = 26 (+2) (Mostly aged between 35 and 54 years. 28 = Female, and no one was identified as an assigned male at birth. Twenty-one per cent were British BAME (African, Indian and Asian). Seventy-five per cent were white British, and 4% identified with gypsy heritage).	Conducted by female academics from Staffordshire University in partnership with Expert Citizens CIC, including female lived experience experts working collaboratively to collect and interpret the data. All researchers had white British heritage, and one had a travelling community background.
3	Online adapted world café with drug and alcohol service and women’s centre professionals.	n = 9 (9 = Female)	Led by female university academics and supported by the wider research team.

## Data Availability

Data are unavailable due to privacy and ethical restrictions.
